# Student-run free clinic volunteers: who they are and what we can learn from them

**DOI:** 10.1186/s12909-021-02793-7

**Published:** 2021-06-26

**Authors:** Fadi W. Adel, Ruth E. Berggren, Robert M. Esterl, John T. Ratelle

**Affiliations:** 1 Department of Internal Medicine, Mayo Clinic, 200 1st St SW, Minnesota 55905 Rochester, United States of America; 2 The Center for Medical Humanities and Ethics, The Joe R. and Teresa Lozano Long School of Medicine, The University of Texas Health Science Center at San Antonio, San Antonio, Texas United States of America; 3 Division of Hospital Internal Medicine, Mayo Clinic, Rochester, Minnesota United States of America

**Keywords:** Student Run Free Clinic, Volunteers, Medical Education, Clinical Skills, Underserved

## Abstract

**Background:**

Initiatives employing medical students’ volunteerism and idealism, such as the Student-Run Free Clinics (SRFC) program, are prevalent in US medical schools. Many studies evaluated various aspects of volunteering, sometimes resulting in conflicting evidence. This study simultaneously sought to identify the characteristics of volunteers vs. non-volunteers, and to characterize the volunteers’ perception of the SRFC.

**Methods:**

We administered a survey to the Long School of Medicine (LSOM) Class of 2018 before their third year of medical school. The authors compared and contrasted the findings of the SRFC volunteers with their non-volunteering counterparts by analyzing their demographics, volunteering history, academic performance, and clinical skills. The volunteers were also asked about their SRFC experiences.

**Results:**

While most volunteers were female (62 %) and non-traditional students (67 %), the difference was not statistically significant (*p* = 0.15 and *p* = 0.38, respectively). Additionally, there were no statistically significant differences between the two groups in measures of academic performance (*p* = 0.25). Most of the volunteers learned about the SRFC program prior to starting medical school. Further, while SRFC volunteers were more likely to engage in additional local volunteering initiatives, the difference was not statistically significant (*p* = 0.03, prespecified  α= 0.006). Importantly, volunteers agreed/strongly agreed that SRFC volunteering emphasized aspects that were missing or underemphasized in the formal medical school curriculum.

**Conclusions:**

Medical students’ age, gender, undergraduate major, and non-traditional status were not statistically different between volunteers vs. non-volunteers. However, there may be tendencies for volunteers to be female, non-traditional, and locally engaged. Further, the timing of knowledge of the SRFC program may not affect student involvement in the SRFC, either. Most importantly, however, while volunteering does not affect the students’ academic performance, it may provide improvements in clinical competencies.

**Supplementary Information:**

The online version contains supplementary material available at 10.1186/s12909-021-02793-7.

## Background

The Liaison Committee on Medical Education lists service learning, an educational experience combining community service with reflection on the students’ roles as citizens and professionals, as one of the core competencies for medical students [1]. Further, the 2010 report of the Carnegie Foundation for the Advancement of Education emphasizes the importance of integration of classroom knowledge with clinical encounters [2]. The Student-Run Free Clinics (SRFC), defined as an initiative where medical students practice real-life history taking, physical exam performing, and clinical decision-making under the supervision of medical faculty in an outpatient setting, are one way to meet these recommendations [3]. The SRFC constitute a prevalent clinical experience among US and Canadian medical schools, and a growing phenomenon internationally [4, 5]. According to a recent study, 106 of the 141 US Association of American Medical Colleges-accredited schools operated 208 SRFC [6]. The majority of medical students at these schools participated in the SRFC, [6, 7] gaining profound benefits academically and professionally.

The educational benefits of volunteering in SRFC are supported by multiple principles of adult learning. For example, the situativity theory argues that knowledge is intimately entwined with the environment and context in which learning occurs [8]. From this perspective, early participation in SFRC may help students appreciate that learning in the classroom is not simply an academic endeavor, but is meant to serve as the foundation of professional knowledge that allows doctors to care for their patients [9]. Having the opportunity to work in an authentic clinical environment helps students understand the context in which knowledge is to be applied and may facilitate future learning.

In addition to the theoretical benefits, evidence from research demonstrates practical benefits of volunteering in SRFC. Participation in the SRFC can expose medical students to core competencies that are underrepresented in some formal curriculum [3, 10, 11]. By working with clinicians from other healthcare professions, medical students can develop an understanding and appreciation of interprofessional collaboration [12, 13]. Volunteering aids in educating students about systems-based practice, a skill necessary for a future physician to navigate the complex healthcare system in order to deliver excellent services to an increasingly diverse patient population [10, 11]. Additionally, involving preclinical students in clinical experiences has a positive impact on their compassion and volunteerism, and students are more likely to become involved in local humanitarian projects [14].

Furthermore, SRFC volunteering may help improve clinical reasoning [15]. While medical students in a classroom organize information based on the structure of the curriculum, clinical professionals demonstrate sound clinical reasoning and possess excellent illness script recognition skills. Therefore, researchers highly recommend that preclinical students be exposed to real-life clinical scenarios, thus improving their pattern-recognition skills, and reinforcing conceptualization rather than memorization [15]. For many preclinical medical students, volunteering at the SRFC provides them with the only opportunity to see real patients [16]. Further, volunteering at an SRFC was associated with a significant, time commitment-dependent improvements in clinical tasks, [3] and that volunteers attained a higher level of academic achievement than their non-volunteering counterparts [17].

Despite the benefits that medical students can amass through participating in the SRFC, not all students volunteer [6]. Indeed, there is uncertainty about which students volunteer and why they choose to volunteer. For example, Blue at al.[18] found a positive association with volunteerism and medical school grade point average. The authors suggest that “students who are stronger academically may find they have more time to provide community service and still maintain their academic success performance.”[18] However, Stoddard et al.[19] found no difference in academic performance between volunteers and their non-volunteering counterparts. To further understand the attributes of SRFC volunteers vs. non-volunteers and describe the experiences of SRFC volunteers, the educational aims of our study were as follows:

1. To compare the characteristics of SRFC volunteers vs. non-volunteers.
2. To measure the experiences and perceptions of SRFC volunteers.

## Methods

### Study design, setting and participants

The LSOM is a public medical school located on the main campus of the University of Texas Health Science Center in San Antonio, Texas (UTHSA). The school trains about 900 medical students and 800 residents annually [20]. The Center for Medical Humanities and Ethics, part of UTHSA, sponsors a multitude of interprofessional community service learning programs, one of which is the SRFC program [21]. All medical students at LSOM are made aware of the volunteering opportunity at the SRFC as part of the admission interview and during orientation prior to starting school. There is no formal expectation to participate in the SRFC (i.e. volunteering in the SRFCs is completely non-mandatory).

The SRFC program at the LSOM encompasses six clinics. The SRFC serve as a student-run, faculty-supervised arena where preclinical first- and second-year medical students practice their classroom-learned clinical skills and apply their textbook knowledge. Student volunteers get the full experience of seeing patients: acquiring consent, taking vitals, obtaining a focused history, performing a relevant physical examination, and discussing those findings with an attending physician. Students work in an interprofessional setting alongside students from other allied health schools, such as nursing students, dental students, and physician assistant students. Additionally, student volunteers are expected to aid in communicating discharge instructions to the patient in a clear and concise manner. Finally, medical student volunteers are expected to document the patient encounter in the Subjective-Objective-Assessment-Plan (SOAP) format and send the note electronically to the attending physician for attestation.

### Survey design and study variables

The survey was developed to serve two purposes: to describe self-reported characteristics of volunteers and non-volunteers, and to outline the experiences of the SRFC volunteers. Therefore, the main author (FWA) delved into literature to learn about commonly asked questions pertaining to medical student demographics, performance, and volunteerism. Subsequently, the survey questions were developed. Next, the second and third authors (REB, RME), who are extensively involved in SRFC administration and medical education, reviewed the survey and provided feedback that resulted in positive adjustments. Then, the survey was uploaded on SurveyMonkey and the link was provided to students.

To assess the demographics of the volunteers (medical students who participated in the SRFC during their 1st and 2nd years of medical school) vs. non-volunteers (medical students who never participated in the SRFC during their 1st and 2nd years of medical school), the survey included questions about the students’ age, gender, undergraduate major(s), traditional vs. non-traditional status. We defined traditional status as students who matriculated in medical school directly after having completed their undergraduate education without any gap years between undergrad and medical schools. Students who did otherwise, whether they pursued another degree, worked, volunteered, took time off to travel or any combination of the aforementioned activities, were considered non-traditional.

To characterize the academic outcomes after volunteering, we included questions regarding the overall preclinical GPA and Clinical Skills module grade. To assess whether the LSOM publicized the SRFC program effectively before orientation, a question regarding first knowledge of SRFC program was incorporated. Further, to identify whether there was any difference in local volunteerism, other than the SRFC program, between volunteers and non-volunteers, we asked the participants whether or not they participated in Frontera de Salud initiatives. Frontera de Salud is a student-run, interdisciplinary volunteering organization with chapters in several medical schools in the state of Texas, USA. Under the supervision of faculty, volunteers provide basic healthcare screenings and education to Spanish-speaking members of the community with limited resources. By cooperating with community partners, students gain skills in cultural competence, health disparities and preventive health. The aforementioned comparisons between volunteers and non-volunteers appear in Table 1.

**Table 1:** Characteristics of Student Volunteers and Non-Volunteers, N (%)

	Volunteer (*n* = 79)	Non-Volunteer (*n* = 26)	p-value
Characteristic			
Age, years			0.27 ^a^
21–25	47 (59.5 %)	17 (65.4 %)	
26–30	27 (34.2 %)	9 (34.6 %)	
> 30	5 (6.3 %)	0	
Gender			0.15 ^b^
Female	49 (62.0 %)	12 (46.1 %)	
Male	30 (38.0 %)	14 (53.8 %)	
Undergraduate Major			0.76 ^a^
Biological Sciences	48 (52.7 %)	16 (50 %)	
Liberal Arts	18 (19.8 %)	5 (15.6 %)	
Natural Sciences (other than Biological Sciences)	6 (6.6 %)	3 (9.4 %)	
Engineering	6 (6.6 %)	1 (3.1 %)	
Other	13 (14.3 %)	7 (21.9 %)	
Traditional Student			0.38 ^b^
Yes	26 (32.9 %)	11 (42.3 %)	
No	53 (67.1 %)	15 (57.7 %)	
GPA			0.25 ^a^
2.5–2.9	7 (9 %)	1 (3.9 %)	
3.0-3.4	34 (43.6 %)	16 (61.5 %)	
3.5-4	37 (47.4 %)	9 (34.6 %)	
When did you learn about the Student-Run Free Clinics (SRFC) program at LSOM?			0.63 ^b^
Before I applied	12 (15.2 %)	5 (19.2 %)	
After I applied but before I was admitted	26 (32.9 %)	6 (23.1 %)	
After I was admitted but before starting school	16 (20.3 %)	4 (15.4 %)	
After I started school	25 (31.6 %)	11 (42.3 %)	
Global Health Elective			0.22 ^b^
Yes	25 (31.6 %)	5 (19.2 %)	
No	54 (68.4 %)	21 (80.8 %)	
Other Volunteering (Frontera de Salud)			0.03 ^b^
Yes	34 (43 %)	5 (19.2 %)	
No	45 (57 %)	21 (80.8 %)	

^a^ Mann-Whitney U test was used

^b^ Chi-square test was used

**Fig. 1:**
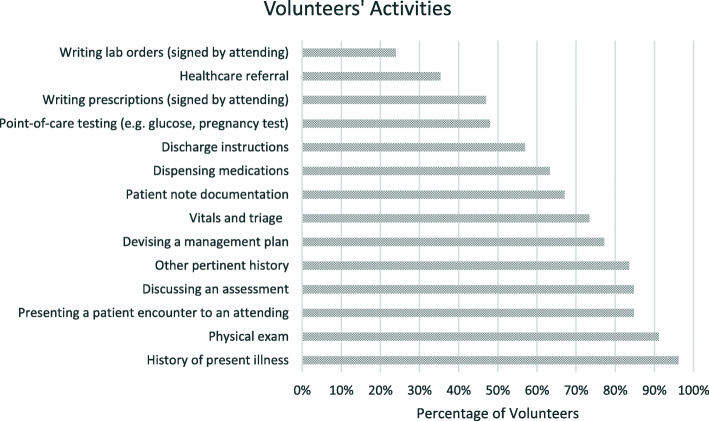
Activities Medical Student Volunteers Participated in at the SRFC

**Fig. 2:**
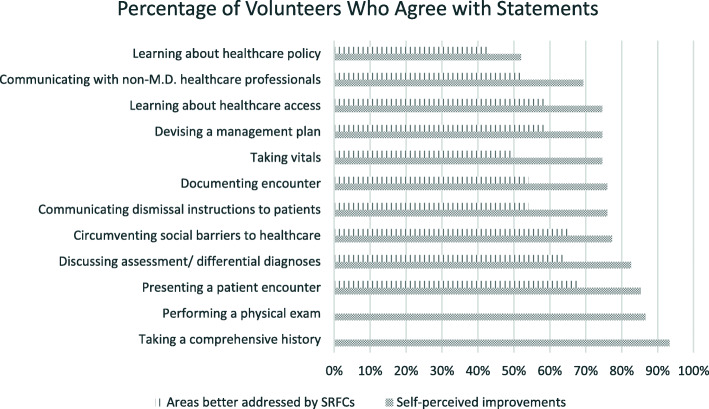
Volunteers’ Self-Reported Improvements in Clinical Skills and Areas Better Addressed Through SRFC Volunteering

To assess the extent of the volunteers’ engagement, their volunteering history, including the frequency of volunteering, the number of patient encounters, and parts of the encounters in which students participated, was explored. Assessment of clinical skills emphasized at the SRFC was also incorporated to evaluate the exact clinical skills being addressed. In order to identify areas that were better addressed by the SRFC than the medical school curriculum, a crucial set of 5-point Likert scale (1- strongly disagree, 5- strongly agree) questions were asked to identify the unique aspect(s) of the SRFC that complemented formal medical education. To assess whether students subjectively experienced progress in clinical skills acquisition, a set of 5-point Likert scale questions regarding self-perceived improvements in clinical skills was incorporated. The data presented in Figs. 1 and 2 pertain to those questions addressed ONLY to volunteers.

### Data collection and analyses

 Our study, which received an exempt status from the Institutional Review Board at LSOM, was conducted through a survey administered online to the LSOM Class of 2018 (Table S1). The survey was administered to students at the end of their pre-clinical years (after the second year of medical school) but before starting their clinical years (third year of medical school). Therefore, volunteers will have had a two-year volunteering time period. The GPA and clinical skills grade were self-reported by the participants. Participation in the survey was anonymous and optional, and it took 10–13 min to complete. Recruitment was performed by the primary author by posting the survey link on the LSOM Class of 2018 Facebook page, as well as by sending the survey link to students through the Facebook messenger. Almost all the students (percentage unknown) in the LSOM class of 2018 were members of the Facebook group as it constituted the main way for the class to communicate announcements and share educational materials. The link to the survey was posted on the Facebook group and was therefore accessible to almost all the students within the class. Additionally, some members of the aforementioned Facebook group were individually contacted through the Facebook messenger by the primary author to encourage them to take the survey.

Accompanying the link to the survey was a statement by the authors indicating the exploratory purpose of the survey, the anonymous nature of the survey, the time length of the survey, the incentive to take survey (ten-dollar Amazon gift cards were given to 10 participants based on a drawing using the participants’ email addresses, the provision of which was optional at the end of the survey), and that, by clicking on the link, the participants consented to taking the survey. Student anonymity was maintained by de-identifying and aggregating all survey responses before data analysis. The survey was conducted in June 2016.

Survey results were summarized using descriptive statistics (percentages, means, modes, medians, standard deviations), as appropriate. Characteristics of student volunteers vs. non-volunteers were compared using Fischer’s exact, Chi-square or Mann-Whitney U tests, as appropriate, and were performed using the Social Science Statistics online calculator (https://www.socscistatistics.com/). Only complete responses were included in the analyses. To account for multiple comparisons, we applied a Bonferroni correction and set a p-value of 0.006 (original *p* = 0.05/8 comparisons between volunteers vs. non-volunteers) for statistical significance.

## Results

### Characteristics of volunteers vs. non-volunteers

The response rate for the survey was 48.6 % (105/216), with the majority of respondents having volunteered at SRFC (*n* = 79, 75.2 %). Characteristics of survey respondents are summarized in Table 1. The majority of students completing the survey were younger than 26 years of age (*n* = 64, 61.0 %), female (*n* = 61, 58.1 %), majored in biological sciences (*n* = 64, 52.0 %) and non-traditional (*n* = 68, 64.8 %). During their time off, 29.3 % of respondents worked or volunteered at a healthcare facility (*n* = 34) and about 17.2 % conducted research (*n* = 20). There were no significant differences in GPA distribution between the two groups (*p* = 0.25).

Additionally, most students learned about the SRFC at UTHSA prior to starting medical school (*n* = 69, 65.7 %). Notably, while student volunteers tended to be more likely to participate in other local outreach programs than non-volunteers, the difference was not statistically significant (*X*^2^ (1, *N* = 105) = 4.75, *p* = 0.03). Further, there were no statistically significant differences between student volunteers and non-volunteers regarding any of the other measured demographic characteristics or academic performance (Table 1).

### Experience of volunteers

Among the 79 SRFC student volunteers, the majority volunteered more than six times during their first two years of medical school (*n* = 45, 56.3 %) and saw more than 10 patients while volunteering (*n* = 46, 58.2 %). There was no significant difference between the median number of patients the volunteers saw during year one (median = 3–4 patients) vs. year two of medical school (median 3–4 vs. 5–6, H (1) = 1.7, *p* = 0.19).

 The most common clinical activities in which volunteers participated were elucidating a patient’s history of present illness (96 %, *n* = 76), performing a physical exam (91 %, *n* = 72), presenting a patient encounter to an attending (85 %, *n* = 67), discussing their assessment (85 %, *n* = 67), devising a management plan (77 %, *n* = 61), and writing a patient note in the SOAP format (67 %, *n* = 53) (Fig. 1).

 Most volunteers participated in activities ranging from obtaining a history to writing a SOAP note and providing discharge instructions. However, less than 50 % participated in performing point-of-care testing, such as capillary glucose checks and urine pregnancy tests.

### Perceptions of volunteers

 Student volunteers agreed/strongly agreed that they experienced improvements in a wide array of core clinical skills as a result of participating in the SRFC program (Fig. 2). Additionally, the majority of volunteers agreed/strongly agreed that participating in the SRFC improved clinical skills that were not highly emphasized during standard medical school clinical skills curriculum.

Not only did volunteers agree/strongly agree that SRFC volunteering helped them improve their clinical skills, but they also agreed/strongly agreed that SRFC volunteering improved their skills in circumventing social barriers to healthcare, interacting with non-MD providers, performing point-of-care tests, and learning about healthcare access and healthcare policy. Additionally, those areas of improvements seemed to overlap with areas better addressed by SRFC volunteering.

 Additionally, most student volunteers (*n* = 63, 85.1 %) agreed/strongly agreed that volunteering at an SRFC helped them improve skills that were not highly emphasized/non-existent in the formal clinical skills course. Areas that were better addressed by SRFC volunteering included presenting a patient encounter, circumventing social barriers to healthcare, discussing assessment/differential diagnoses, and devising a plan (Fig. 2). Lastly, the majority of respondents (*n* = 62, 81.6 %) agreed/strongly agreed that volunteering at an SRFC should be integrated into the preclinical curriculum.

## Discussion

The SRFC address important aspects of medical education. Understanding the characteristics of students who do vs. do not volunteer may help identify barriers to volunteering and inform future interventions to foster participation in SRFC. In this single-center study, there were no major statistically significant differences between SRFC volunteers and non-volunteers in terms of demographics or academic performance, yet volunteers tended to be female and non-traditional. In the SRFC, volunteers mainly participated in obtaining history, performing physical exams, discussing assessment and plan, and documenting encounters. Consequently, the majority of volunteers indicated self-perceived improvements in the aforementioned areas. Most importantly, the overwhelming majority of volunteers agreed that the SRFC emphasized areas not highly emphasized/addressed in the formal clinical curriculum, and that the SRFC should be integrated into the preclinical curriculum.

The fact that there were no statistically significant differences between the volunteers and their non-volunteering counterparts in terms of demographics, undergraduate major, or being traditional medical students hints at the possibility of other, putative drivers of volunteerism. While not statistically significant, the differences in GPA and gender between volunteers and non-volunteers (Table 1) may be relevant. Also, despite lack of statistical significance, there seems to be a signal that volunteers were more likely to engage in local volunteering opportunities (Frontera de Salud), which is consistent with findings in the literature [12]. Additionally, there was no difference between the two groups in terms of their involvement in global health initiatives, indicating that local, rather than global, community service may be an important driving factor in the engagement of volunteers in the SRFC. A future study that involves more participants and crosses multiple medical schools may be able to shed further light on these differences.

The majority of respondents in both groups learned about the SRFC program before staring medical school, which suggests that early awareness alone is insufficient to promote student volunteerism. This finding, in combination with the similar demographics between volunteers and non-volunteers, raises an important question: what motivates some students, but not others, to participate in SRFC? There are likely to be many answers to this question, as motivation in medical education is a complex construct [22]. However, identifying the barriers and facilitators to volunteerism is an important direction of future research to ensure that students, and their patients, reap the full benefits of SRFC.

 Remarkably, despite the lack of measurable differences in academic performance between the two groups, volunteers consistently agreed/strongly agreed that their SRFC involvement provided them with a set of skills that was not highly emphasized or non-existent in the formal medical school curriculum. This suggests that medical schools should consider integrating the SRFC program as a non-graded component of the undergraduate medical curriculum. With that approach, students will have the opportunity to experience the unique educational benefits of taking care of patients at the SRFC without having to be concerned about receiving a grade that impacts their GPA. Also, the majority of volunteers agreed/strongly agreed that they experienced self-perceived betterment in learning about healthcare access and circumventing social barriers, which is consistent with prior literature [12].

We found that there was no difference in academic performance between the volunteers and non-volunteers as measured by preclinical GPA. This is in contrast with Blue et al. [16] and Vaikunth et al.[17], who found that community service involvement correlated with better GPA, but it is consistent with Stoddard et al. [17]. The inconsistency may be due to the fact that we only measured preclinical GPAs, and we may be missing the potential differences in GPAs experienced in clinical years. Regardless, our findings support existing literature that volunteering at SRFC does not interfere with students’ preclinical classroom performance.

## Limitations

The sample in this study comes from one school, which may impact the applicability of the data. Further, volunteers comprised the majority of respondents. This might have been due to sampling bias, since we utilized convenience sampling methods. This resulted in a low number of non-volunteers, perhaps affecting the statistical significance of some of our findings. Moreover, the participant responses were not individually tracked to help with data stratification. Furthermore, only preclinical data was collected, which might impair the assessment of the impact on the participants’ skills during the clinical years of medical education and beyond. Lastly, the students’ GPA was used as a measure of academic performance, which, while objective, does introduce its own bias into the results.

## Conclusions

In this single-center study, there were no major statistically significant differences between SRFC volunteers and non-volunteers in terms of demographics or academic performance, yet volunteers tended to be female and non-traditional. Additionally, the majority of all students were made aware of SRFC opportunities prior to entering medical school, implying unmeasured influences of volunteerism. Given the myriad of benefits to learning and patient care, future research efforts should focus on identifying and overcoming barriers to participation in SRFC. Additionally, given the self-reported benefits of the SRFC, serious consideration should be given to incorporating them further into the medical curriculum.

## Supplementary information

**Additional file 1**

## Data Availability

The datasets used and/or analyzed during the current study are available from the corresponding author on reasonable request.

## Abbreviations

SRFC: Student-Run Free Clinics
UTHSA: The University of Texas Health Science Center in San Antonio, Texas
LSOM: The Long School of Medicine
SOAP: Subjective-Objective-Assessment-Plan
GPA: Grade Point Average

## References

[1] The Liaison Committee on Medical Education (LCME). FUNCTIONS AND STRUCTURE OF A MEDICAL SCHOOL- Standards for Accreditation of Medical Education Programs Leading to the M.D. Degree. 2012.

[2] Irby D, Cooke M, O’Brien B (2010). Calls for Reform of Medical Education by the Carnegie Foundation for the Advancement of Teaching: 1910 and 2010. Academic Medicine.

[3] Stephens L, Bouvier N, Thomas D, Meah Y (2015). Voluntary Participation in a Medical Student-Organized Clinic for Uninsured Patients Significantly Augments the Formal Curriculum in Teaching Underrepresented Core Competencies. J Stud Run Clin.

[4] Drexler R, Fröschle F, Predel C, Sturm B, Ustorf K, Lehner L, Janzen J, Valentin L, Scheer T, Lehnert F, Tadzic R, Oldhafer KJ, Meyer TN (2020). Establishing a student-run free clinic in a major city in Northern Europe: a 1-year experience from Hamburg, Germany. J Public Health (Oxf).

[5] Fröberg M, Leanderson C, Fläckman B, Hedman-Lagerlöf E, Björklund K, Nilsson GH, Stenfors T. Experiences of a student-run clinic in primary care: a mixed-method study with students, patients and supervisors. Scand J Prim Health Care. 2018 Mar;36(1):36–46. 10.1080/02813432.2018.1426143 PMC5901439 29368978

[6] Smith S, Thomas R, Cruz M, Griggs R, Moscato B, Ferrara A (2014). Presence and Characteristics of Student-Run Free Clinics in Medical Schools. JAMA.

[7] Gorrindo P, Peltz A, Ladner T, Reddy I, Miller B, Miller RF, Fowler MJ (2014). Medical Students as Health Educators at a Student-Run Free Clinic: Improving the Clinical Outcomes of Diabetic Patients. Acad Med.

[8] Durning SJ, Artino AR. “Situativity theory: a perspective on how participants and the environment can interact: AMEE Guide no. 52.“. Med Teach. 2011;33(3):188–99. 10.3109/0142159X.2011.550965 21345059

[9] Maudsley G, Strivens J (2000). Promoting professional knowledge, experiential learning and critical thinking for medical students. Med Educ.

[10] Sheu L, O’Brien B, O’Sullivan PS, Kwong A, Lai CJ (2013). Systems-Based Practice Learning Opportunities in Student-Run Clinics: A Qualitative Analysis of Student Experiences. Acad Med.

[11] Meah YS, Smith EL, Thomas DC (2009). Student-Run Health Clinic: Novel Arena to Educate Medical Students on Systems-Based Practice. Mt Sinai J Med.

[12] Holmqvist M, Courtney C, Meili R, Dick A (2012). Student-Run Clinics: Opportunities for Interprofessional Education and Increasing Social Accountability. Journal of Research in Interprofessional Practice Education.

[13] Pammett R, Landry E, Weidmann AE, Jorgensen D (2015). Interprofessional student-run primary health care clinics: Educational experiences for pharmacy students. CPJ/RPC.

[14] Smith JK, Weaver DB (2006). Capturing Medical Students’ Idealism. Ann Fam Med.

[15] Bowen JL (2006). Educational Strategies to Promote Clinical Diagnostic Reasoning. N Engl J Med.

[16] Xu J. Letting Medical Students Run The Clinic. The Atlantic. 2013; Accessed 2/20/18. https://www.theatlantic.com/health/archive/2013/11/letting-medical-students-run-the-clinic/281241/.

[17] Vaikunth SS, Cesari WA, Norwood KV, Satterfield S, Shreve RG, Ryan JP, Lewis JB (2014). Academic Achievement and Primary Care Specialty Selection of Volunteers at a Student-Run Free Clinic. Teach Learn Med.

[18] Blue AV, Geesey ME, Sheridan ME, Basco WT (2006). Performance outcomes associated with medical school community service. Acad Med.

[19] Stoddard HA, Risma JM (2011). Relationship of participation in an optional student-run clinic to medical school grades. Teach Learn Med.

[20] UT Health San Antonio-Long School of Medicine. (2020). About US. https://www.uthscsa.edu/academics/medicine/about.

[21] Center for Medical Humanities and Ethics-UT Health San Antonio. About Us. https://www.texashumanities.org/about/.

[22] Cook DA, Artino AR (2016). Motivation to learn: an overview of contemporary theories. Med Educ.

