# Supplementary material for: Student-run free clinic volunteers: who they are and what we can learn from them
Source: BMC Med Educ. 2021 Jun 26;21:356. doi: 10.1186/s12909-021-02793-7 (PMC8236183; doi:10.1186/s12909-021-02793-7)
Supplement: Supplementary file 1 — Additional file 1 [file 12909_2021_2793_MOESM1_ESM.docx]

Supplemental Table 1- Survey Administered to Students Between Second and Third Years of Medical School

Demographics:

1. Age Group: A- 21-25 B- 26-30 C-31-35 D- 36-40 E- 41-45 F- 46-50 G- >50
2. Gender:
3. Male
4. Female
5. Other
6. Undergraduate Major (S): Select all that apply
7. Architecture
8. Business
9. Communications
10. Education
11. Engineering
12. Fine Arts
13. Geosciences
14. Liberal Arts
15. Biological Sciences
16. Natural Sciences (other than Biological Sciences)
17. Undeclared
18. Other:

- Please, specify:

1. Are you a traditional medical student (i.e. did you enter medical school immediately following your undergraduate education?)
2. Yes
3. No
4. If you answered No, please specify what you did in your gap year(s): Select all that apply
5. Worked/Volunteered at a healthcare facility
6. Conducted research
7. Worked/Volunteered at a non-healthcare facility
8. Traveled
9. Pursued an advanced degree
10. Other: - please, specify
11. Your current medical school GPA:
12. <2.5
13. 2.5-2.9
14. 3-3.4
15. 3.5-4
16. My overall grade in the Clinical Skills module is:
17. A
18. B
19. C
20. F
21. What field were you mostly interested in when you started medical school? (Rank your top 3 choices)
22. Allergy and Immunology
23. Anesthesiology
24. Cardiothoracic Surgery
25. Colon and Rectal Surgery
26. Dermatology
27. Emergency Medicine
28. Family Medicine
29. Geriatrics
30. Internal Medicine
31. Internal Medicine-Subspecialty
32. Internal Medicine-Pediatrics
33. Medical Genetics
34. Neurological Surgery
35. Neurology
36. Nuclear Medicine
37. Obstetrics and Gynecology
38. Ophthalmology
39. Orthopedic Surgery
40. Otolaryngology
41. Pathology
42. Pediatrics
43. Physical Medicine and Rehabilitation
44. Plastic Surgery
45. Preventative Medicine
46. Psychiatry
47. Radiation Oncology
48. Radiology-Diagnostic
49. Sleep Medicine
50. Surgery-General
51. Surgery-Subspecialty
52. Urology
53. Other

- Please, specify:

1. When did you learn about the Student-Run Free Clinics (SRFC) program at UTHSCSA SOM?
2. I did not know about it before applying
3. I learned about it after I applied
4. I learned about it after I was admitted but before starting school
5. I learned about it after I started school

Longitudinal Preceptorship

1. How many visits did you have with your UTHSCSA SOM-assigned Longitudinal Preceptor?
2. >9
3. 9
4. 5-8
5. 1-4
6. How many hours did you spend cumulatively with your preceptor?
7. < 20 hours
8. 20-30 hours
9. 30-40 hours
10. > 40 hours
11. What was your preceptor’s primary specialty of practice?
12. Allergy and Immunology
13. Anesthesiology
14. Cardiothoracic Surgery
15. Colon and Rectal Surgery
16. Dermatology
17. Emergency Medicine
18. Family Medicine
19. Geriatrics
20. Internal Medicine
21. Internal Medicine-Subspecialty
22. Internal Medicine-Pediatrics
23. Medical Genetics
24. Neurological Surgery
25. Neurology
26. Nuclear Medicine
27. Obstetrics and Gynecology
28. Ophthalmology
29. Orthopedic Surgery
30. Otolaryngology
31. Pathology
32. Pediatrics
33. Physical Medicine and Rehabilitation
34. Plastic Surgery
35. Preventative Medicine
36. Psychiatry
37. Radiation Oncology
38. Radiology-Diagnostic
39. Sleep Medicine
40. Surgery-General
41. Surgery-Subspecialty
42. Urology
43. Other

Please, specify:

1. Having been part of the UTHSCSA SOM Longitudinal Preceptorship program, please indicate your level of agreement with each of the following statements. Consider each statement starting with ***“I believe that I have improved in… due to my longitudinal preceptorship”***

| 1. Activity | Not Applicable | Strongly Disagree | Disagree | Neutral | Agree | Strongly Agree |
| --- | --- | --- | --- | --- | --- | --- |
| Taking vitals |  |  |  |  |  |  |
| Taking history and HPI |  |  |  |  |  |  |
| Performing physical exam |  |  |  |  |  |  |
| Presenting patient encounter |  |  |  |  |  |  |
| Discussing assessment/ differential diagnoses |  |  |  |  |  |  |
| Devising a management plan |  |  |  |  |  |  |
| Running Tests (e.g. pregnancy, glucose) |  |  |  |  |  |  |
| Ordering labs |  |  |  |  |  |  |
| Prescribing meds |  |  |  |  |  |  |
| Dispensing meds |  |  |  |  |  |  |
| Referring to specialists |  |  |  |  |  |  |
| Communicating discharge instructions to patients |  |  |  |  |  |  |
| Documenting encounter |  |  |  |  |  |  |
| Learning about healthcare policy |  |  |  |  |  |  |
| Learning about healthcare access |  |  |  |  |  |  |
| Recognizing illness scripts |  |  |  |  |  |  |
| Circumventing social barriers to healthcare |  |  |  |  |  |  |
| Communicating with non-M.D. healthcare professionals |  |  |  |  |  |  |

Volunteering History

1. Have you ever volunteered at any of the SRFC at UTHSCSA?
2. Yes
3. No
4. Approximately, how many times have you volunteered over the past two years?
5. 1-2
6. 2-4
7. 5-7
8. 8-10
9. > 10
10. Approximately, how many times did you volunteer as a first year medical student?
11. 1-2
12. 2-4
13. 5-7
14. 8-10
15. > 10
16. Approximately, how many times did you volunteer as a second year medical student?
17. 1-2
18. 2-4
19. 5-7
20. 8-10
21. > 10
22. At which of the following SRFCs did you volunteer? ( Select all that apply)
23. SAMM
24. Haven For Hope
25. Alpha Home
26. Refugee Clinic
27. Travis Park Dermatology Clinic
28. Mobile Eye Clinic
29. How many patients have you seen (individually, or as part of a team of student volunteers) overall during your visits to the SRFCs?
30. 0-3
31. 4-6
32. 7-10
33. 10-13
34. >14
35. In which part(s) of the patient encounter did you partake at a SRFC? (Select all that apply)
36. Vitals and triage
37. History of present illness
38. Other pertinent history
39. Physical exam
40. Presenting a patient encounter to an attending
41. Discussing an assessment
42. Devising a management plan
43. Running tests (e.g. glucose levels, pregnancy tests, dipstick urinalysis)
44. Writing lab orders
45. Writing prescriptions
46. Dispensing medications
47. Healthcare referral
48. Discharge instructions
49. Patient note documentation
50. Did you participate in Frontera de Salud events?
51. Yes
52. No
53. If you answered Yes to question 17, how many hours did you invest in Frontera de Salud?
54. <10
55. 10-20
56. 20-30
57. 30-40
58. >40
59. Did you participate in the Global Health Elective at UTHSCSA, or have you ever been on a medical mission trip?
60. Yes
61. No
62. If you answered Yes to question 19, approximately how many hours did you spend doing volunteer work?
63. < 40 hours
64. 40-60 hours
65. 61-80 hours
66. 81-100 hours
67. > 100 hours
68. Having volunteered at the UTHSCSA SOM SRFC program, please indicate your level of agreement with each of the following statements. Consider each statement starting with ***“I believe that I have improved in… due to being involved in the SRFC program”***

| Activity | Not Applicable | Strongly Disagree | Disagree | Neutral | Agree | Strongly Agree |
| --- | --- | --- | --- | --- | --- | --- |
| Taking vitals |  |  |  |  |  |  |
| Taking history and HPI |  |  |  |  |  |  |
| Performing physical exam |  |  |  |  |  |  |
| Presenting patient encounter |  |  |  |  |  |  |
| Discussing assessment/ differential diagnoses |  |  |  |  |  |  |
| Devising a management plan |  |  |  |  |  |  |
| Running Tests (e.g. pregnancy, glucose) |  |  |  |  |  |  |
| Ordering labs |  |  |  |  |  |  |
| Prescribing meds |  |  |  |  |  |  |
| Dispensing meds |  |  |  |  |  |  |
| Referring to specialists |  |  |  |  |  |  |
| Communicating discharge instructions to patients |  |  |  |  |  |  |
| Documenting encounter |  |  |  |  |  |  |
| Learning about healthcare policy |  |  |  |  |  |  |
| Learning about healthcare access |  |  |  |  |  |  |
| Recognizing illness scripts |  |  |  |  |  |  |
| Circumventing social barriers to healthcare |  |  |  |  |  |  |
| Communicating with non-M.D. healthcare professionals |  |  |  |  |  |  |

1. Do you believe that volunteering at a SRFC has helped you improve skills that are not highly emphasized/non-existent in the formal UTSHCSA SOM Clinical Skills course (NOT INCLUDING the Longitudinal Preceptorship part)?
2. Yes
3. No
4. Maybe
5. If you answered Yes or Maybe, please indicate which area(s) was (were) better addressed by volunteering at an SRFC than learning through the formal Clinical Skills (NOT INCLUDING the Longitudinal Preceptorship part)? Select all that apply:
6. Vitals and triage
7. History of present illness
8. Other pertinent history
9. Physical exam
10. Presenting patient encounter to an attending
11. Discussing assessment
12. Devising a management plan
13. Running tests (e.g. glucose levels, pregnancy tests, dipstick urinalysis)
14. Writing lab orders
15. Writing prescriptions
16. Dispensing medications
17. Healthcare referral
18. Discharge instructions
19. Patient note documentation
20. Recognizing illness scripts
21. Recognizing social barriers to healthcare
22. Helping patients gain access to healthcare
23. Other

- Please, specify:

1. Do you believe that volunteering at a SRFC has helped you improve skills that are not highly emphasized/non-existent in the formal UTSHCSA SOM Longitudinal Preceptorship?
2. Yes
3. No
4. Maybe
5. If you answered Yes or Maybe, please indicate which area(s) was (were) better addressed by volunteering at an SRFC than learning through the Longitudinal Preceptorship? Select all that apply:
6. Vitals and triage
7. History of present illness
8. Other pertinent history
9. Physical exam
10. Presenting patient encounter to an attending
11. Discussing assessment
12. Devising a management plan
13. Running tests (e.g. glucose levels, pregnancy tests, dipstick urinalysis)
14. Writing lab orders
15. Writing prescriptions
16. Dispensing medications
17. Healthcare referral
18. Discharge instructions
19. Patient note documentation
20. Recognizing illness scripts
21. Recognizing social barriers to healthcare
22. Helping patients gain access to healthcare
23. Other

- Please, specify:

1. How were the SRFC improvements gauged? Select all that apply:
2. Self-perceived improvements
3. Standardized patient feedback
4. Clinical Skills course grade
5. Clinical Elective
6. Other

- Please, specify:

1. What field(s) are you most interested in as of now? (Rank your top 3 choices)
2. Allergy and Immunology
3. Anesthesiology
4. Cardiothoracic Surgery
5. Colon and Rectal Surgery
6. Dermatology
7. Emergency Medicine
8. Family Medicine
9. Geriatrics
10. Internal Medicine
11. Internal Medicine-Subspecialty
12. Internal Medicine-Pediatrics
13. Medical Genetics
14. Neurological Surgery
15. Neurology
16. Nuclear Medicine
17. Obstetrics and Gynecology
18. Ophthalmology
19. Orthopedic Surgery
20. Otolaryngology
21. Pathology
22. Pediatrics
23. Physical Medicine and Rehabilitation
24. Plastic Surgery
25. Preventative Medicine
26. Psychiatry
27. Radiation Oncology
28. Radiology-Diagnostic
29. Sleep Medicine
30. Surgery-General
31. Surgery-Subspecialty
32. Urology
33. Other

- Please, specify:

1. Having volunteered at an SRFC, please indicate your level of agreement with each of the following statements:

| Statement | Not Applicable | Strongly Disagree | Disagree | Neutral | Agree | Strongly Agree |
| --- | --- | --- | --- | --- | --- | --- |
| The SRFC experience affected my future specialty choice |  |  |  |  |  |  |
| I am more likely to seek a primary-care specialty in the future |  |  |  |  |  |  |
| I am more likely to partake in public health initiatives |  |  |  |  |  |  |
| I am more confident in my clinical skills |  |  |  |  |  |  |
| The SRFC experience made me feel more prepared going into clinical years of my medical education |  |  |  |  |  |  |
| I am more confident during my standardized patient encounters |  |  |  |  |  |  |
| I find myself better at linking classroom knowledge with patient encounters |  |  |  |  |  |  |
| I feel more confident taking patients’ histories |  |  |  |  |  |  |
| I feel more prepared performing focused physical exams |  |  |  |  |  |  |
| I feel more confident communicating patients’ findings to attendings |  |  |  |  |  |  |
| I feel more prepared recognizing illness scripts |  |  |  |  |  |  |
| I feel more confident ordering labs |  |  |  |  |  |  |
| I feel more confident ordering meds |  |  |  |  |  |  |
| I feel more confident documenting patients’ encounters |  |  |  |  |  |  |
| I feel more confident going into my clinical years |  |  |  |  |  |  |
| I feel more comfortable in outpatient settings |  |  |  |  |  |  |
| The SRFC was my only way of seeing real patients outside my longitudinal preceptorship |  |  |  |  |  |  |
| I have a better understanding of health equity issues |  |  |  |  |  |  |
| Volunteering prompted me to think of ways to address those inequities |  |  |  |  |  |  |
| I have a better appreciation of interprofessional work environments |  |  |  |  |  |  |
| I have improved my foreign language skills |  |  |  |  |  |  |
| I had trouble signing up for an available spot to volunteer at an SRFC |  |  |  |  |  |  |
| I believe volunteering at an SRFC should be integrated into the pre-clinical curriculum |  |  |  |  |  |  |

Thank you for your participation in this survey.
